# Health need assessment in an indigenous high-altitude population living on an island in Lake Titicaca, Perú

**DOI:** 10.1186/s12939-019-0993-3

**Published:** 2019-06-18

**Authors:** María Calderón, Rosa Alvarado-Villacorta, Miguel Barrios, Devy Quiroz-Robladillo, Doris Rocío Guzmán Naupay, Ana Obregon, Sthefanny Calderón Chávez, Lisa Glaser, Andres M. Carnero, Carla Cortez-Vergara, David Iglesias Quilca, Jose Colque Gonzales, David Moore

**Affiliations:** 1 London School of Hygiene and Tropical Medicine, London, UK; 2 Universidad Peruana Cayetano Heredia, Lima, Peru; 3 Hospital Nacional Cayetano Heredia, Lima, Peru; 4 Natural System and Life Care NGO, Lima, Peru; 5 Universidad Particular Inca Garcilaso de la Vega, Lima, Peru; 6 TARGET, Public Health England, London, UK; 7 GSK Vaccines, Lima, Peru; 8 Universidad Nacional de Ingenieria, Lima, Peru

**Keywords:** Amantani, Peru, Health need assessment, Indigenous

## Abstract

**Background:**

Health needs and access to health care is a huge challenge in developing countries, especially in some isolated indigenous communities. Amantani is an island located at 3854 m above sea level in Lake Titicaca, Peru. There is no official date on key local health needs and determinants, which precludes the prioritization and efficient implementation of health interventions. The objective of this study is to validate a health need assessment tool and ascertain the main health needs of the indigenous high-altitude population living on Amantani.

**Methods:**

We conducted a cross-sectional study to describe the health needs of the indigenous population of Amantani using a questionnaire based on the “Peruvian Demographic and Health Survey”. The questionnaire underwent expert and field-work validation. We selected a random sample of the island residents using two-stage cluster sampling. We estimated the prevalence of key health needs and determinants, and evaluated their distribution by age, sex and education through prevalence ratio. All analyses accounted for the complex sampling design.

**Results:**

We surveyed 337 individuals (223 adults and 144 children) in 151 houses. The most frequent health needs were: (i) lack of access to medical screening for a)non-communicable diseases (> 63.0%) and b)eye problems (76.5%); and (ii) poor knowledge about communicable diseases (> 54.3%), cancer (71.4%) and contraception (> 32.9%). Smoking and alcohol use was more frequent in males (PR = 4.70 IC95%:1.41–15.63 and PR = 1.69 95% CI:1.27–2.25, respectively). People with higher education had more knowledge about TB/HIV and cancer prevention (*p* < 0.05). Regarding children’s health, > 38% have never undergone eye or dental examination. Corporal punishment and physical bullying at school in the last month were relatively common (23 and 33%, respectively).

**Conclusion:**

The main health needs in Amantani are related to poor healthcare access and lack of awareness of disease prevention. Our findings can be used to develop and implement efficient health interventions to improve the health and quality of life of indigenous populations living in the islands in Southern Peru/Northern Bolivia.

**Electronic supplementary material:**

The online version of this article (10.1186/s12939-019-0993-3) contains supplementary material, which is available to authorized users.

## Background

Amantani is one of the islands located in Lake Titicaca in the region of Puno, in the southern end of Peru. The island has an area of 9.28 km^2^ and is located at an altitude of 3854 m above sea level in its lowest parts. According to the 2007 national census, Amantani has a total of 4255 inhabitants. The main language spoken in the area is Quechua. The majority of the population live in rural areas (72.3% of the island area) and the main source of income is agriculture and tourism [1]. Amantani is one of the poorest districts in the region of Puno, with 93.8% of the population living in poverty, and 55% living in extreme poverty [1].

Amantani can only be reached by boat. The trip usually takes between 2 and 4 h from the nearest towns (Capachica or Puno). The island has only one primary health facility that offers primary health care in general medicine, dentistry and nursing [2]. Medical care is provided by a general practitioner from the rural service program (which is mandatory for new graduates seeking to apply for medical specialty training), and the position is on a yearly rotation. Free medical care can be accessed through the Integral Health Insurance, although registration has to be made in the city of Puno [2]. When laboratory testing is required, samples have to be transported to the city of Puno for analysis, taking approximately 1 to 2 weeks for the results to be returned to the patient [3]. If specialized care is indicated, patients are referred to the Hospital of Puno, although this does not ensure immediate care. Upon referral, the patient has to pay for all of the required expenses (including transport to the hospital) [3].

Given the unique cultural, socioeconomic and geographic characteristics of Amantani, we pondered upon how such determinants may have impacted the health of the population. After conducting a comprehensive search, we found no relevant information from scientific literature or official reports. The only available source of potentially-relevant information was the Demographic and Family Health Survey (ENDES, in Spanish Encuesta Demográfica y de Salud Familiar) [4], an annual national survey (last results available from 2014) that has information on sociodemographic and health indicators in the region of Puno, and has regional, departmental and national representativeness. However, the survey was not stratified by district, and does not allow establishing the specific health indicators of the population of Amantani. Furthermore, no surveys were performed in the islands within Lake Titicaca. The distinctive cultural and social characteristics of Amantani makes it difficult to extrapolate information from the data available for Puno.

## Methods

### Setting and population

The cross-sectional study was performed in Amantani Island in Puno, Peru. All the people who answered the surveys had to meet the following eligibility criteria: a) Residents of Amantani (including adults and children) who live in the selected household b) At least more than 50% of the people in the household spoke Spanish. Those who refused, did not have the capacity to give informed consent to participate or were unable answer the questionnaire in the study were excluded.

### Tool and validation process

We developed and validated a questionnaire to measure the health needs of the population of Amantani. Our instrument was based on the ENDES national survey [4]. It collected information on demographic, socioeconomic and health indicators of women of reproductive age (15–49 years) and their children under five residing in Peru. Its results have regional, departmental and national representativeness. ENDES is a Peruvian adaptation of the Demographic Health Survey that has been used in over 80 developing countries for over 30 years [5]. For the sake of this study, the section of women’s reproductive health was excluded for male participants.

After these adjustments, we performed two types of qualitative validation: a) expert validation and b) field validation. The first one, was performed by seven Peruvian health professionals with experience working in rural settings, who specialized in internal medicine, ophthalmology, infectious diseases, dentistry, reproductive health, public health and mental health. They reviewed all questions with special emphasis on their specialties. After collecting all of the comments and suggestions, the questionnaire was modified accordingly and sent back to the experts for their approval.

For field validation, we decided to conduct 10 health surveys in three households using a convenient sample. We made sure to interview at least one female, one male, an older person and a child. After finishing the health need questionnaire, we asked questions related to feasibility, relevance, and acceptability of the survey. We tried to establish whether or not the respondents felt comfortable answering the questions, there was an adequate understanding of the questions and the questionnaire duration was acceptable. Moreover, we asked if they felt that answering these questions was going to help the authors to accomplish the study objective, and if they had any suggestions for improving the survey.

It is important to emphasize that the assessment of the prevalence of diabetes and hypertension was based on self-report. No clinical procedures or laboratory tests were requested or performed for confirming the diagnosis. In the case of children under 5 years of age, questions were asked to their mothers and included queries related to pregnancy, antenatal care, delivery, the postpartum period, and breastfeeding.

The validated instrument had two levels of administration: household and individual. The dimensions of the questionnaire are as follows: a) Household Level: people living in the household, disability, water: availability, sources, quality of drinking water, sanitation, house material characteristics; and b) Individual Level: non-communicable diseases and risk factors, eye health, dental care, mental health, prevention of oncologic disease, obstetric care: prenatal, delivery, postnatal, knowledge and the symptoms and the transmission of tuberculosis, knowledge about the transmission of HIV/AIDS (Human immunodeficiency virus infection/Acquired immune deficiency syndrome), contraception: knowledge and practice.

### Sampling

We conducted a two-stage cluster sampling, in which we first sampled households (clusters), and then individuals within households. Given that we were interested in multiple outcomes with unknown and wide-ranging prevalence, we assumed a conservative prevalence of 50% for the sample size calculation, in order to attain sufficient precision for all outcomes (as this results in the maximum sample size). In addition, we assumed a margin of error for the prevalence of 5%, and a confidence level of 95%. Finally, we assumed a population size of 4255 [1], and the average household size of four inhabitants in accordance to the last national census.

Following the methodology of Bennet et al. [6] for sample size calculation for clustered samples, and using the parameters described above, we obtained a sample size of approximately 150 households (clusters). Thereby, the desired precision for estimating the health needs in Amantani required sampling a minimum of 150 houses.

### Selection of households

We used simple random sampling to select households using Google Earth. Then, using a random number generator, we randomly selected 150 households to be included in the study. Subsequently, we identified the coordinates of each of these houses, and created a geographic layer with their location. This layer was uploaded through Google maps to all of the mobile devices that were going to be used in the fieldwork. This allowed each interviewer to have precise directions to locate the selected households. A printed map was also available for areas with limited GPS access, as we knew in advance that several areas of the island had poor internet and telephone connectivity.

In cases where the selected households were abandoned or had no residents present at the time of the visit, we selected a replacement household from neighbouring houses according to a predefined random rule. Moreover, 22 households (12.4%) refused to participate in the survey and 4 household were excluded because they speak only in quechua (2.26%).

### Data collection

The data collection was held in July 2017. We started the recruitment of volunteer fieldworkers through Facebook approximately 1 month beforehand. During the application process, we prioritized individuals with experience in community work in the health field. After selection, we were able to recruit nine volunteers (doctors, public health specialist, biologist, dentist and one medical student). Each of them were given a mobile phone with the questionnaires and maps of selected households downloaded and underwent two training sessions and a final test.

An informed consent was obtained from each participant. Only houses that met the study selection criteria were included. The individual-level questions were personally asked to the each of the adults living in the household, while parents/tutors answered the questions about the children. In the case of adolescents between 12 and 17 years, they could answer the questions provided they had the consent of their parents. All the data recollected was stored on each mobile phone and sent to a matrix database when Internet access was available.

### Data analysis

We used Stata 14 Data Analysis and Statistical Software for data analysis, which was conducted both at the household and individual level. We summarized categorical variables using a relative frequency analysis while also presenting absolute values. For continuous variables, we used the arithmetic mean as a measure of central tendency, and the standard deviation as a measure of dispersion. For the bivariate and regression analysis, all numerical variables were categorised. For the bivariate analysis, we used the chi-square test. We chose to model the prevalence ratio (PR) directly using a Poison Model, as opposed to using the odds ratio as an approximation, because the odds ratio overestimates the prevalence ratio when the outcome is not uncommon (> 10%) [7]. Analyses took into account the characteristics of the survey design (clustering, sampling weights) by using the survey estimation commands (svy) in Stata.

## Results

### Instrument validation

#### Expert validation

Expert validation of the questionnaire was performed by Peruvian health professionals specialized in internal medicine, ophthalmology, infectious diseases, dentistry, reproductive health, public health and mental health. Experts unanimously agreed on the need to shorten the questionnaire from 1000 questions originally to 120 questions (100% agreement).

Questions about diet were simplified by only including questions about consumption of fruits and vegetables. The ENDES originally measured the consumption of various types of food, like juices, fruit salads, dressings, etc., which was irrelevant to this population in Amantani according to the opinion of our experts.

Regarding eye health, questions about access to eye care were added for the diagnosis and treatment of cataract. Also, we added questions regarding the reasons why a child did not use prescribed glasses. In the reproductive health section, questions about the ideal number of children per family were added.

Many questions were eliminated from the questionnaire due to being irrelevant to the study objectives (< 20% of the original questions). In the case of mental health, questions about domestic violence were specifically eliminated because the expert considered that, given its sensitive nature, it required special training of data collectors, and access to a private and safe environment for women during the interview.

#### Field validation

Field validation was conducted five days before the fieldwork commenced and the results were analysed qualitatively. In terms of acceptability, the questionnaire was regarded positively by the population. Respondents said that it could help to raise awareness of the authorities regarding the current health care conditions which could ultimately lead to improvements of health services available in the island. Respondents were willing to invest their time answering the questionnaire and the duration of the questionnaire was not considered unduly long.

We found that the questions about contraception were not well understood by women. Upon further discussion, we realized that they had some knowledge about several contraceptive methods but did not recognize the names included in the questionnaire. For this reason, we supplemented this section of the questionnaire with pictures of the contraceptive methods.

In addition, we perceived that many men felt threatened by the questions about the amount of alcohol consumed. Many of them told us that they did not think questions about alcohol consumption were important because they were only “social drinkers” and not “alcoholics”. Given the discomfort caused by these questions, and their secondary relevance to the study objectives, we decided not to include them and only ask if the person had drink alcohol in the last month.

Other questions that were eliminated included those concerning suicidal ideation, as they made the villagers uncomfortable. These topics could be assessed in future studies with personnel specialized in these subjects, and adequate private spaces. Final version of the questionnaire in Spanish is shown Additional file 1.

## Main health needs

In the 151 houses included in the study, we surveyed 337 individuals (223 adults and 144 children). Sociodemographic characteristics of the population (Table 1) showed that 26.9% of the participants have never received formal education as compared to only 2.7% receiving higher education (university or technical studies). Moreover, we observed a high number of participants that are currently registered in the Peruvian national insurance of health (more than 80%).

**Table 1:** Demographic characteristics of adults and children

Characteristics	N (%)
**Adults (*N*** **=** **223)**	
Age (Mean, SD)	46.58 (17.71)
Female	132 (59.2)
Highest education	
Never went to school	60 (26.9)
Incomplete Primary	39 (17.5)
Complete Primary	37 (16.6)
Incomplete Secondary	33 (14.8)
Complete Secondary	48 (21.5)
Superior	6 (2.7)
National insurance	185 (83)
**Children (*N*** **=** **144)**	
Age (Mean, SD)	8.17 (4.7)
Female	71 (49.3)
School attendance	
Pre-schoolers	26 (18.1)
Currently attending	118 (8.2)
National insurance	139 (96.5)

Regarding housing conditions, we found that most of the houses had either no floor (49%) or cement floor (19%); adobe walls (89.4%); and calamine roofs (98.7%). The main lighting source was solar panels (65%). However, almost 16% of houses relied on candlelight for illumination. 74.8% of households reported using firewood for cooking and 82.8% had a chimney (Table 2).

**Table 2:** Self- Reported Household General Characteristics (*N* = 151)

Household General Characteristics	N (%)
Number of people per household	
Children	
0	56 (37.1)
1	37 (24.5)
2	34 (22.5)
≥ 3	24 (15.9)
Adults	
1	21 (13.9)
2	78 (51.7)
3	37 (24.5)
≥ 4	15 (9.9)
Disabilities present in at least one resident	
To move	23 (15.2)
To see	34 (22.5)
To hear	25 (16.6)
To talk	13 (8.6)
To understand	20 (13.3)
Fuel used for cooking	
Firewood	113 (74.8)
Liquid gas	5 (3.3)
Firewood and gas	27 (17.9)
Manure	1 (0.7)
Charcoal	5 (3.3)
Presence of a chimney	125 (82.8)
Space in which to cook	
Inside the house	61 (40.4)
Unroofed area	7 (4.6)
Separate room	83 (55)
Type of Lighting	
Solar Panel	98 (65)
Candle	24 (15.9)
Battery	18 (11.9)
Other	11 (7.3)
Floor Material	
Natural Ground Floor	74 (49.0)
Cement	29 (19.2)
Stone	22 (14.6)
Other	26 (17.2)
Wall Material	
Adobe	135 (89.4)
Other	16 (10.6)
Ceiling Material	
Calamine	149 (98.7)
Reinforced concrete	2 (1.3)

Summary of the main health needs are (Table 3) showed that more than 75% of the adults interviewed did not have glucose measurement in the last year and had never had an eye examination. In women, more than 70% had never heard about cancer, or had any type of screening (mammography or pap smear). Regarding contraceptive methods, 67% had never hear about the morning-after pill, and 70.6% had never used contraception. As for children, the most common health need was related to never having an eye examination. Notably, more than 20% of the children had experienced corporal punishment or physical bullying at school in the last month.

**Table 3:** Summary of most frequent Health Needs in Amantani

Characteristics	N	%
Adults		
Non-communicable diseases and risk factors		
Did not have a blood pressure measurement in the last year (*N* = 216)	136	63.0
Did not have a glucose measurement in the last year (*N* = 209)	174	83.3
Consumed fruits less than twice per week (*N* = 222)	152	68.5
Eye health		
Never had an eye examination (*N* = 234)	179	76.5
Difficulty seeing/recognising a face within 6 m in the last year (*N* = 234)	104	44.4
Communicable diseases		
Had never heard about tuberculosis (N = 223)	121	54.3
Had never heard about HIV/AIDS (*N* = 230)	156	67.8
Oral health		
Never had a dental examination (*N* = 232)	84	36.2
Never had information about dental care (*N* = 232)	131	56.5
Mental Health		
Self-report of anhedonia or sadness (*N* = 226)	153	67.7
Knowledge or Cancer Prevention		
Had never heard about cancer (*N* = 105)	75	71.4
Never had a mammography (*N* = 105)	75	71.4
Never had a pap smear (*N* = 105)	82	78.1
Contraception		
Had never heard about fallopian tubal ligation (*N* = 85)	57	67.1
Had never heard about oral contraceptive pills (*N* = 85)	39	45.9
Had never heard about the male condom (*N* = 85)	28	32.9
Had never heard about the morning-after pill (*N* = 85)	57	67.1
Hadnever used contraception (*N* = 85)	60	70.6
Children		
Eye health		
Never had an eye examination (*N* = 63)	29	46.0
Inadequate lighting forreading (*N* = 44)	16	36.4
Oral health		
Never had a dental examination (*N* = 122)	47	38.5
Have never had information about dental care (*N* = 122)	41	33.6
Mental health		
Corporal punishment by a teacher in the last month (*N* = 46)	11	23.9
Physical violence by another student in the last month (*N* = 46)	15	33.3

We performed an analysis of the main health needs by age, gender and level of education. Analysis of women health needs will be addressed in another publication. Given that from the public health point of view reporting unadjusted results is more useful, we will refer to the unadjusted results even though we also performed adjusted results described as prevalence ratios (Tables 4, 5 and 6). However, it is important to highlight that there was no significant difference between these two different analysis approaches. Moreover, we have included the percentage of health needs divided by age, gender and level of education in Additional file 2.

**Table 4:** Multivariable analysis between health needs and gender

Characteristic	Crude PR (95% CI)		Adjusted^a^ PR (95% CI)	
	Female	Male	Female	Male
Adults				
Non-communicable diseases and risk factors				
Blood pressure measurement in the last year	Ref.	0.86 (0.59–1.24)	Ref.	0.83 (0.58–1.18)
Self-report of a diagnosis of high blood pressure	Ref.	0.75 (0.23–2.45)	Ref.	0.73 (0.22–2.35)
Smoking in the last year	Ref.	7.04 (2.83–17.53)	Ref.	6.75 (2.66–17.10)
Smoking in the last month	Ref.	4.89 (1.51–15.86)	Ref.	4.70 (1.41–15.63)
Lifetime prevalence of alcohol use	Ref.	1.67 (1.25–2.23)	Ref.	1.69 (1.27–2.25)
Alcohol use in the last month	Ref.	1.60 (0.83–3.09)	Ref.	1.56 (0.81–2.99)
Fruit consumption in the last week	Ref.	0.96 (0.88–1.05)	Ref.	0.97 (0.88–1.06)
Eye health				
Lifetime prevalence of visual acuity testing	Ref.	1.67 (1.06–2.62)	Ref.	1.76 (1.09–2.83)
Difficulty seeing/recognising a face within 6 m in the last year	Ref.	1.02 (0.78–1.32)	Ref.	0.95 (0.75–1.19)
Communicable diseases				
Heard about tuberculosis	Ref.	1.17 (0.94–1.45)	Ref.	1.23 (1.00–1.52)
Heard about HIV/AIDS	Ref.	1.00 (0.69–1.44)	Ref.	1.27 (0.91–1.78)
Depression				
Self-report of anhedonia or sadness	Ref.	0.75 (0.53–1.05)	Ref.	0.84 (0.62–1.13)
Children				
Eye health				
Lifetime prevalence of visual acuity testing	Ref.	0.91 (0.60–1.39)	Ref.	N/A
Self-report of a diagnosis of visual impairment or prescription of glasses	Ref.	2.92 (0.57–14.88)	Ref.	N/A

^a^ Adjusted by age

**Table 5:** Multivariable analysis between health needs and age

Characteristic (among adults)	Crude PR (95% CI)			Adjusted^a^ PR (95% CI)		
	Young adults (18–34 yrs)	Middle-aged adults (35–59 yrs)	Older adults (≥60 yrs)	Young adults (18–34 yrs)	Middle-aged adults (35–59 yrs)	Older adults (≥60 yrs)
Non-communicable diseases and risk factors						
Blood pressure measurement in the last year	Ref.	0.85 (0.52–1.38)	1.32 (0.83–2.10)	Ref.	0.86 (0.53–1.39)	1.36 (0.85–2.17)
Self-report of a diagnosis of high blood pressure	Ref.	1.08 (0.26–4.45)	1.48 (0.33–6.70)	Ref.	1.10 (0.26–4.57)	1.55 (0.34–7.05)
Smoking in the last year	Ref.	1.27 (0.52–3.07)	2.06 (0.79–5.38)	Ref.	1.10 (0.47–2.57)	1.55 (0.60–4.02)
Smoking in the last month	Ref.	1.06 (0.25–4.39)	1.87 (0.41–8.52)	Ref.	0.93 (0.22–3.89)	1.47 (0.33–6.65)
Lifetime prevalence of alcohol use	Ref.	1.27 (0.84–1.92)	0.94 (0.59–1.50)	Ref.	1.21 (0.80–1.84)	0.86 (0.54–1.36)
Alcohol use in the last month	Ref.	2.05 (0.65–6.43)	0.64 (0.17–2.46)	Ref.	1.97 (0.62–6.20)	0.62 (0.16–2.32)
Fruit consumption in the last week	Ref.	0.91 (0.81–1.03)	0.88 (0.75–1.02)	Ref.	0.91 (0.81–1.03)	0.88 (0.75–1.03)
Eye health						
Lifetime prevalence of visual acuity testing	Ref.	0.35 (0.19–0.65)	0.57 (0.30–1.09)	Ref.	0.33 (0.18–0.62)	0.52 (0.28–0.98)
Difficulty seeing/recognising a face within 6 m in the last year	Ref.	1.93 (1.11–3.36)	3.91 (2.33–6.55)	Ref.	1.94 (1.11–3.37)	3.94 (2.36–6.60)
Communicable diseases						
Heard about tuberculosis	Ref.	0.93 (0.70–1.23)	0.52 (0.32–0.86)	Ref.	0.91 (0.69–1.21)	0.50 (0.31–0.82)
Heard about HIV/AIDS	Ref.	0.50 (0.34–0.74)	0.16 (0.07–0.38)	Ref.	0.49 (0.33–0.73)	0.15 (0.06–0.37)
Cancer						
Knowledge of cancer prevention (among women)	Ref.	0.64 (0.35–1.18)	N/A	Ref.	0.72 (0.40–1.29)	N/A
Depression						
Self-report of anhedonia or sadness	Ref.	0.95 (0.64–1.42)	0.97 (0.6–1.56)	Ref.	0.96 (0.65–1.43)	1.00 (0.62–1.60)

^a^ Adjusted by sex

**Table 6:** Multivariable analysis between health needs and education

Characteristic (among adults)	Crude PR (95% CI)			Adjusted^a^ PR (95% CI)		
	Primary school	Secondary school % (95% CI)	Higher education	Primary school	Secondary school % (95% CI)	Higher education
Non-communicable diseases and risk factors						
Blood pressure measurement in the last year	1.07 (0.65–1.76)	Ref.	1.94 (0.91–4.12)	1.05 (0.58–1.91)	Ref.	2.10 (0.96–4.60)
Self-report of a diagnosis of high blood pressure	1.06 (0.15–7.46)	Ref.	10.43 (1.69–64.32)	0.60 (0.02–21.57)	Ref.	10.58 (1.5–74.86)
Smoking in the last year	1.61 (0.61–4.26)	Ref.	6.53 (2.31–18.47)	2.84 (0.94–8.60)	Ref.	7.10 (2.05–24.65)
Smoking in the last month	1.71 (0.42–7.02)	Ref.	7.62 (1.49–39.05)	1.93 (0.27–13.86)	Ref.	7.92 (0.84–74.66)
Lifetime prevalence of alcohol use	0.94 (0.67–1.33)	Ref.	1.50 (0.88–2.58)	0.91 (0.59–1.39)	Ref.	1.37 (0.79–2.40)
Alcohol use in the last month	1.01 (0.39–2.62)	Ref.	1.16 (0.16–8.25)	0.77 (0.24–2.46)	Ref.	0.92 (0.10–8.73)
Fruit consumption in the last week	0.95 (0.84–1.08)	Ref.	0.92 (0.64–1.34)	0.94 (0.78–1.14)	Ref.	0.96 (0.67–1.36)
Eye health						
Lifetime prevalence of visual acuity testing	0.50 (0.28–0.90)	Ref.	0.87 (0.25–3.00)	0.73 (0.34–1.57)	Ref.	1.34 (0.37–4.78)
Difficulty seeing/recognising a face within 6 m in the last year	1.36 (0.88–2.09)	Ref.	1.39 (0.56–3.49)	0.72 (0.46–1.13)	Ref.	1.09 (0.41–2.90)
Communicable diseases						
Heard about tuberculosis	0.69 (0.51–0.93)	Ref.	1.08 (0.73–1.59)	0.62 (0.45–0.86)	Ref.	1.01 (0.68–1.48)
Heard about HIV/AIDS	0.48 (0.31–0.76)	Ref.	1.99 (1.17–3.41)	0.85 (0.50–1.46)	Ref.	1.72 (1.01–2.93)
Cancer						
Knowledge of cancer prevention (among women)	0.54 (0.24–1.23)	Ref.	3.43 (1.27–9.23)	0.46 (0.20–1.05)	Ref.	2.38 (1.57–3.60)
Depression						
Self-report of anhedonia or sadness	0.69 (0.44–1.07)	Ref.	1.01 (0.29–3.47)	0.82 (0.48–1.41)	Ref.	0.71 (0.21–2.39)

^a^ Adjusted by age and sex

Alcohol consumption in the last month was more frequent in men, as compared to women. In addition, smoking was notably more reported in males, being almost seven times more prevalent than females. Other differences found between both genders were that men had more knowledge regarding tuberculosis and that they had undergone more visual acuity testing as compared to women. More details in Table 4.

Looking at different age categories within adults, it was more frequent for young adults to have had a visual acuity testing in the last year (40.0%) compared to middle-age and older adults (13.0 and 21.1% respectively). Difficulties recognizing faces at 6 m were more frequent in older adults. Also, we found that age was inversely associated with the knowledge about tuberculosis, HIV/AIDS and cancer prevention. More details shown in Table 5.

Self-reporting of high blood glucose, high blood pressure or smoking was more frequent among individuals with higher education. Interestingly, individuals with secondary school (33.0%) had a higher frequency of visual acuity testing or eye examination than subjects with higher education (28.6%). Nevertheless, both frequencies were higher than that among individuals with primary education (16.5%). As expected, education was directly associated with awareness about tuberculosis, HIV/AIDS and cancer prevention. More details are shown in Table 6.

## Discussion

This study describes the main health needs of Amantani by using a locally validated instrument in a random sample of the island settlers. The main health needs in the island were: lack of access to medical screening for non-communicable diseases and eye health; and poor knowledge about communicable diseases, cancer and contraception.

Unfortunately there was scarce information about validated health needs assessment tools in the literature, particularly for developing countries. One of the tools we found was designed by the World Health Organization for use in Europe at a community level [8]. This tool had many similar dimensions to our instrument (quality of drinking water, behaviour and lifestyle, socioeconomic status based on the household material and basic services, access to health and individual health). Nevertheless, the tool included different items like a) social environment, defined as the degree of social and emotional support receive from friends and/or family and b) family genetics [8]. Another technical document was designed by the National Institute for Clinical Excellence for use at a national and regional level in the United Kingdom [3]. This tool included additional dimensions regarding homelessness, refugee status and ethnicity, culture and sexuality. Most of these additional dimensions, although valuable for the specific context in which they were administered, were not relevant for our study population (e.g. refugee status; and homelessness and ethnicity, which do not vary within the island), prohibitively costly (family genetics), and/or overly sensitive (family genetics, cultural issues, sexuality, social support). Regarding the latter, sensitive topics have to be assessed adopting a strategic, specialized approach that was not possible to develop in this particular project. This is the reason why we have not included more in-depth questions about alcohol use or domestic violence. We, however, believe this has to be complemented in future versions of our tool.

The most frequent health needs were the lack of access to medical screening for a) non-communicable diseases (blood pressure, glucose measurement) b) eye examination and c) oral care. This is similar to previous reports in indigenous populations [9, 10] including other regions of Peru [11]. According with our results, more than 80% of the population have access to the Peruvian national insurance, hence all this services should be covered. Regarding children’s health, more than 38% never had an eye or dental exam. We noticed that the health access for children was better than that for adults. This is in agreement with other studies where adults reported that they prefer to seek medical attention for their children rather than for themselves [12].

Poor knowledge of communicable diseases (> 54.3%), cancer (71.4%) and contraception (> 32.9%) was also common. These results contradicted findings from the official ENDES 2017 report. When the same determinants were reviewed, Puno was among the regions with the most knowledge about the prevention of these diseases [13, 14]. This might show that despite the efforts of the region to promote health awareness, it has been a huge challenge reaching populations in these isolated territories. In the analysis by education, as expected, people with higher education had more knowledge about TB/HIV and cancer prevention.

Alcohol use was more frequently stated in males compared to female inhabitants. However, specific inferences about this cannot be made since other important determinants could not be measured. This included the amount units consumed on a certain occasion, frequency of drinking and the ability to stop drinking alcohol, among others. Likewise, tobacco use was notably more frequent in male than females.

Corporal punishment and physical bullying at school in the last month were relatively common (23 and 33%, respectively). Other studies have reported the presence of bullying in schools in Brazil [15] and in Lima (Peru) [16] but it has never been described in an island population. Official reports regarding this matter have not been published for Puno.

An important strength of our study is the fact that the instrument had two different validation processes (including thematic experts and the population “local experts”) that ensured the validity and robustness of the questionnaire, providing a holistic perspective of the local health needs which combined the views of the health specialists and the settlers. This type of cross-cultural validation is crucial for adequately measuring local health issues especially in indigenous populations, and has been previously used in different parts of the world with translation to the indigenous language [17–19]. In our case, although pictures helped to improve the understanding of the questions, we may have had higher response rates (> 85.4%) if the questionnaire had been in Quechua. However, the questionnaire applied without problems in the houses (in some cases with the aid of a fieldworker who spoke Quechua or family members), and very few houses were excluded due to a language barrier.

One important limitation of the study is that the questionnaire could not confirm the diagnoses as it was based on self-reporting. We believe that this questionnaire should be supplemented with auxiliary tests such as blood glucose measurement or glycated haemoglobin, and blood pressure measurement to allow a more accurate estimation of the true prevalence of hypertension and diabetes. Another limitation was the short period of time for the study, mainly due to lack of funding. However, we believe that this work lays the foundation for a series of more in-depth studies in the island that will be expand the health needs assessment tool, quantify the prevalence of health needs in a more in-depth manner, and implement and evaluate the impact of simple, yet efficient, health interventions.

Although our tool has limitations as we previously mentioned, the results have shed some light on the health situation in Amantani. For example, it was observed in a general way that some determinants differ from those reported in official national documents such as knowledge of communicable diseases and contraception which was recorded as high in the Puno region. This is an important finding that can help health decision makers strengthen their efforts in the dissemination of this knowledge on the island. Moreover, we have found out that the vast majority of the population was covered by national insurance, nonetheless the access to health services seemed to be quite limited. The barriers that could be hindering services from reaching remote areas like Lake Titicaca need to be addressed by health authorities in Peru.

Other strengths of this study are the representativeness of the sample (which may be extrapolated to other islands in Lake Titicaca), its novelty (regarding the study population), and the direct applicability of its findings. Special emphasis was placed on the calculation of the sample by clusters so that the results have a good internal and external validity. Random sampling using geographic tools allowed selecting a fairly well-distributed sample across the island.

## Conclusions

This study is one of the first approaches to measure the health needs of populations living in the islands in Lake Titicaca in Southern Peru and Northern Bolivia.

We are convinced that this document is an important tool for health decision makers to develop and implement targeted interventions that prioritize the most important health needs in Amantani. Finally, the validated tool developed as part of this study can be used in other islands in Lake Titicaca, and although it could certainly be expanded and adapted to the specific target population, the tool still includes many of the important health indicators and allows performing a general evaluation of health needs.

## Additional files

Additional file 1: Health Needs Questionnaire (Spanish Original Version). (DOCX 56 kb)

Additional file 2: Complementary bivariate analysis of health needs. (DOCX 24 kb)

## Data Availability

The datasets used and/or analysed during the current study are available from the corresponding author on reasonable request.
