# Supplementary material for: Health need assessment in an indigenous high-altitude population living on an island in Lake Titicaca, Perú
Source: Int J Equity Health. 2019 Jun 18;18:94. doi: 10.1186/s12939-019-0993-3 (PMC6582488; doi:10.1186/s12939-019-0993-3)
Supplement: Supplementary file 1 — Health Needs Questionnaire (Spanish Original Version). (DOCX 56 kb) [file 12939_2019_993_MOESM1_ESM.docx]

**SUPPLEMENTARY MATERIAL 1: Questionnaire (Spanish Original Version)**

It is important to emphasize that the original interview was carried out in an electronic format that allowed to pass from one question to another depending on whether the answer was positive or negative. Although the questionnaire seems extensive, there were cases in which they could last 5 minutes according to the person who answered and their specific answers.

| Questions | Possible answers | Sources |
| --- | --- | --- |
| **DISCPACIDAD EN EL HOGAR** |  |  |
| A continuación le haré algunas preguntas para saber si en su casa vive alguna persona con limitación o dificultad PERMANENTE, que le impida o le dificulte desarrollarse normalmente en sus actividades cotidianas.  A) Para moverse, caminar, usar sus brazos y/o piernas?  B) Para ver, a pesar de usar lentes o anteojos?  C) Para oir, aun usando audífonos?  D) Para hablar o comunicarse?  E) Para entender o aprender (concentrarse y recordar)?  F) Tiene alguna otra limitación de manera permanente? | Si  No  No sabe | Un miembro de la familia que tenga el mayor conocimiento sobre el tema (sólo se realizará una vez). |
| **ABASTECIMIENTO DE AGUA** |  |  |
| ¿Cuál es la principal fuente de abastecimiento de agua que utilizan en su hogar para tomar o beber? | RED PUBLICA  Dentro de la vivienda  Fuera de la vivienda, pero dentro del edificio  Pilón /grifo público  AGUA DE POZO  Pozo en la casa/patio/lote  Pozo público O Manantial (puquio)  Río / acequia / laguna  Agua de Lluvia  Camión Tanque/Aguatero  Agua embotellada | Un miembro de la familia que tenga el mayor conocimiento sobre el tema (sólo se realizará una vez). |
| ¿La fuente de abastecimiento de agua en el hogar está disponible todo el día? | Si  No | Un miembro de la familia que tenga el mayor conocimiento sobre el tema (sólo se realizará una vez). |
| En las últimas dos semanas. ¿Tuvo un corte de agua todo un día o más tiempo? | Si  No | Un miembro de la familia que tenga el mayor conocimiento sobre el tema (sólo se realizará una vez). |
| En su hogar, ¿Habitualmente toman o beben agua tal como viene del caño, pozo o fuente de abastecimiento? | Tomar tal como viene de la fuente  La hierven  Le echan lejía/ cloro  Utiliza un filtro de agua especial  Filtrar a través de una tela  Desinfección solar  Dejar reposar  Toman agua embotellada  Otro | Un miembro de la familia que tenga el mayor conocimiento sobre el tema (sólo se realizará una vez). |
| ¿El agua que usan para beber, es agua que conservan en un envase o recipiente? | Si  No | Un miembro de la familia que tenga el mayor conocimiento sobre el tema (sólo se realizará una vez). |
| ¿Lo usa con una tapa? | Si  No | Un miembro de la familia que tenga el mayor conocimiento sobre el tema (sólo se realizará una vez). |
| **SERVIVIO HIGIÉNICO/ HIGIENE/ DESECHO DE BASURA** |  |  |
| ¿Qué tipo de servicio de higiénico tiene su hogar?  (Para esta pregunta se utilizará ayuda gráfica) | CONECTADO A LA RED PUBLICA   - Dentro de la vivienda - fuera de la vivienda, pero dentro del edificio - Fosa séptica / fosa séptica   LETRINA   - Mejorada ventilada - Mejorada ecológica/abonera/compostera - Mejorada colgante/flotante - ¿Pozo ciego o negro con tratamiento de cal, ceniza, estiércol, aserrín o arena? - Pozo ciego o negro - Río, acequia o canal - No hay servicio_________ - Otro ____ | Un miembro de la familia que tenga el mayor conocimiento sobre el tema (sólo se realizará una vez). |
| El servicio higiénico es de uso exclusivo del hogar? | Si  No | Un miembro de la familia que tenga el mayor conocimiento sobre el tema (sólo se realizará una vez). |
| ¿Con qué frecuencia limpian o realizan el aseo del baño o letrina? | Varias veces al día  Una vez al día  Varias veces por semana  Una vez por semana  Cada 8 o más días  nunca  No se | Un miembro de la familia que tenga el mayor conocimiento sobre el tema (sólo se realizará una vez). |
| Los miembros de este hogar, ¿Dónde se lavan las manos? | Dentro o cerca baño  Dentro o cerca de la cocina  En otro lugar dentro de la vivienda  Fuera de la vivienda  No hay un lugar específico. | Un miembro de la familia que tenga el mayor conocimiento sobre el tema (sólo se realizará una vez). |
| Ustedes, cómo eliminan o dónde botan la mayor parte de la basura orgánica; es decir la piel de tubérculos, frutas, verduras, restos de alimentos preparados y / o desechos del jardín? | Se recoge en la casa (gobierno, municipio, comunidad, instituciones privadas)  Contenedores (gobierno, municipio, comunidad, comunidad, instituciones privadas)  Depositado o arrojado  Campo abierto  En la calle  En lugar distante  dentro del patio o terreno  río / zanja  Quemado  Enterrado  Compost  Comida para animales  Otro _ | Un miembro de la familia que tenga el mayor conocimiento sobre el tema (sólo se realizará una vez). |
| ¿Cada qué tiempo recogen la basura? | diario  Varias veces a la semana  Semanal  Cada 15 días  Uno por mes  Frecuencia irregular  Otro ______  No se | Un miembro de la familia que tenga el mayor conocimiento sobre el tema (sólo se realizará una vez). |
| Antes de tirar la basura ¿En qué la almacena? | Recipiente o contenedor cubierto  Recipiente o contenedor descubierto  Bolsa de plástico/ costal de polietileno /yute  Bolsa de papel / periódico/caja de cartón  No tiene recipiente  Otro  No sabe | Un miembro de la familia que tenga el mayor conocimiento sobre el tema (sólo se realizará una vez). |
| **COMBUSITBLES** |  |  |
| ¿Cuál es el combustible más usado en su hogar para cocinar? | Electricidad  Gas líquido  Gas natural (tubería)  Kerosene  Carbón vegetal  Carbón mineral  Leña  Estiércol/Bosta  Residuos agrícolas  Cañas / Arbustos secos  No cocinar  Otros | Un miembro de la familia que tenga el mayor conocimiento sobre el tema (sólo se realizará una vez). |
| En esta casa, la cocina, fogón o estufa en la que preparan sus alimentos, ¿Tiene una chimenea o conducto para eliminar el humo? | Si  No | Un miembro de la familia que tenga el mayor conocimiento sobre el tema (sólo se realizará una vez). |
| Generalmente, ¿se cocina dentro de la casa, al aire libre o en un cuarto separado? | Dentro de la casa  Aire libre / patio / terraza  Cuarto separada |  |
| ¿Utilizas un cuarto especialmente para cocinar? | Si  No | Un miembro de la familia que tenga el mayor conocimiento sobre el tema (sólo se realizará una vez). |
| ¿Qué tipo de iluminación usas en tu casa? | Electricidad  Gas llicuado (GLP)  Gas natural (tubería)  Kerosene  Velas  Batería  Otros | Un miembro de la familia que tenga el mayor conocimiento sobre el tema (sólo se realizará una vez). |
| **MATERIAL PREDOMINANTE EN VIVIENDA** |  |  |
| Material predominante en el piso de la vivienda | Piso terminado  Parquet o madera pulida  Láminas asfálticas, vinílicas o similares  losetas, terrazas o similares  Cemento / ladrillo  Piso rústico de madera  Piso rústico de Pona  Piso natural de tierra/arena  Otro | Un miembro de la familia que tenga el mayor conocimiento sobre el tema (sólo se realizará una vez). |
| Material predominante de las paredes exteriores de la vivienda | Ladrillo o bloque de cemento  Piedra o sillar con cal o cemento  Adobe o "tapia tarrajeados"  Rústicos  Adobe o "tapia sin tarrajear"  tablones/madera  Quincha (caña con barro  Piedra con barro  Natural / ligero  Varilla / bambu / pona / palma / troncos / tabique  Estera  Cartón  Tripley  Sin paredes  Otros | Un miembro de la familia que tenga el mayor conocimiento sobre el tema (sólo se realizará una vez). |
| Material predominante del techo de la vivienda | Preparado / terminado  Concreto armado  Tejas  Rústicos  Placa de calamina, fibra de cemento o similar  Madera  caña o estera con torta de barro  Natural / ligero  Paja, hojas de palmera, etc.  Estera  Cartón  Sin techo  Otro | Un miembro de la familia que tenga el mayor conocimiento sobre el tema (sólo se realizará una vez). |
| Tipo de ventanas de la vivienda | No tiene ventanas  Ventanas con vidrio  Ventanas con madera  Ventanas con malla  Ventanas con cortinas o persianas | Un miembro de la familia que tenga el mayor conocimiento sobre el tema (sólo se realizará una vez). |
| **HIPERTENSIÓN/DIABETES** |  |  |
| En el último año, ¿un médico u otro personal de salud midió su presión arterial? | Si  No  No sé/ No recuerda | Miembros del hogar mayores de 15 años |
| ¿Dónde se midió su presión arterial la última vez? | Hospital publico  Centro/puesto de salud publico  Hospital de Essalud  Policlínico/posta de Essalud  Fuerzas armadas / Policía  Clínica/consultorio privado  Clínica/posta de ONG  Hospital de iglesia  Campaña del ministerio  Campaña de Essalud  Otras campañas  Otros  No sé / No recuerdo | Miembros del hogar mayores de 15 años |
| ¿Su médico le diagnosticó alguna vez "hipertensión arterial" o "presión alta"? | Si  No  No se/ No recuerda | Miembros del hogar mayores de 15 años |
| ¿Hace cuánto tiempo le dijeron a usted que tiene hipertensión arterial o "presión alta"? | En "meses", si <2 años  En "años", si 2 o más años  No sé / no recuerda | Miembros del hogar mayores de 15 años |
| En el último año,¿ha recibido y / o comprado medicamentos para controlar su presión arterial? | Si  No  No se/ No recuerda | Miembros del hogar mayores de 15 años |
| ¿ En el último año, recibió o compró sus medicamentos con receta médica al menos una vez? | Con receta médica  Ninguna receta médica  No sé/ No recuerdo | Miembros del hogar mayores de 15 años |
| ¿ En el último año, tomó sus medicamentos según las indicaciones del médico? | Si  No  No sé/ No recuerda | Miembros del hogar mayores de 15 años |
| En el último año, ¿algún médico u otro personal de salud midió su glucosa en la sangre o "azúcar" en la sangre? | Si  No  No sé/ No recuerda | Miembros del hogar mayores de 15 años |
| ¿Dónde le midieron la glucosa o el "azúcar" en la sangre la última vez?  Considere el lugar donde le midieron la glucosa y no el lugar donde le indicaron la prueba | Hospital publico  Centro/puesto de salud publico  Hospital de Essalud  Policlínico/posta de Essalud  Fuerzas armadas / Policía  Clínica/consultorio privado  Clínica/posta de ONG  Hospital de iglesia  Campaña del ministerio  Campaña de Essalud  Otras campañas  Otros  No sé / No recuerdo | Miembros del hogar mayores de 15 años |
| ¿Alguna vez un médico ha diagnosticado diabetes o "alto nivel de azúcar en la sangre" en su vida? | Si  No  No recuerda | Miembros del hogar mayores de 15 años |
| ¿Hace cuánto tiempo le han dicho que usted tiene diabetes o "azúcar alta” en la sangre"? | Si  No  No recuerda | Miembros del hogar mayores de 15 años |
| En el último año, ¿ha recibido y / o comprado medicamentos para controlar su "diabetes" o "azúcar alta” en la sangre"? | Si  No  No recuerda | Miembros del hogar mayores de 15 años |
| ¿ En el último año, recibió o compró sus medicamentos recetados al menos una vez? | Con receta médica  Ninguna receta médica  No sabe/ No recuerda | Miembros del hogar mayores de 15 años |
| ¿ En el último año, tomó sus medicamentos según las indicaciones del médico?  Considere como "sí" cuando el participante manifiesta inyección de insulina | Si  No  No recuerda | Miembros del hogar mayores de 15 años |
| **FACTORES DE RIESGO DE ENFERMEDADES NO TRASNMISIBLES** |  |  |
| En el último año, ¿ha fumado cigarrillos? | Si  No  No recuerda | Miembros del hogar mayores de 15 años |
| En el último mes, ¿ha fumado cigarrillos? | Si  No  No recuerda | Miembros del hogar mayores de 15 años |
| ¿Fuma a diario? | Si  No  No recuerda | Miembros del hogar mayores de 15 años |
| ¿Cuántos años tenía cuando empezó a fumar diariamente? | Edad | Miembros del hogar mayores de 15 años |
| ¿Hace cuantos años empezó a fumar diariamente? | Años | Miembros del hogar mayores de 15 años |
| Normalmente, ¿cuántos cigarrillos fuma cada día? | Número de Cigarrillos  No recuerda | Miembros del hogar mayores de 15 años |
| ¿Ha consumido alguna vez en su vida alguna bebida alcohólica o licor? | Si  No  No recuerda | Miembros del hogar mayores de 15 años |
| ¿Cuántos años tenía la primera vez que bebió licor? | Edad | Miembros del hogar mayores de 15 años |
| En el último año y mes ¿ha consumido alguna bebida alcohólica o licor? | Si  No  No recuerda | Miembros del hogar mayores de 15 años |
| En el último mes, ¿cuántas veces bebió alguna bebida alcohólica o licor? | Número de veces | Miembros del hogar mayores de 15 años |
| De esas_______ veces que tomó, piense en la vez que consumió más, y dígame. Qué bebida alcohólica tomó? | - Cerveza - Vino / cachina / champagne - Chicha de jora fermentada - Masato Fermentado - Yonque / cañazo - Anisado - Whisky / pisco / ron - Otros - No recuerdo / No sé | Miembros del hogar mayores de 15 años |
| En la última semana, ¿cuántos días comió fruta? Si es que ha comido. | Número de días  No comió  No sé / No recuerdo | Miembros del hogar mayores de 15 años |
| ¿Cuántas unidades, tajadas o racimos de frutas comió por día? | Número de unidades  No sé / No recuerdo | Miembros del hogar mayores de 15 años |
| En la última semana, ¿cuántos días bebió jugo de fruta? Si es que ha tomado  (Incluye extractos de frutas) | Número de días  No tomó  No sé / No recuerdo | Miembros del hogar mayores de 15 años |
| ¿Cuántos vasos de jugo de fruta tomó por día?  En la última semana, ¿cuántos días comiste ensalada de frutas? Si es que ha comido | Número de vasos  No sé / No recuerdo | Miembros del hogar mayores de 15 años |
| ¿Cuántas porciones de ensalada de frutas comió por día? | Número de porciones  No sé / No recuerdo | Miembros del hogar mayores de 15 años |
| En la última semana, ¿cuántos días comiste ensalada de vegetales? Si ha comido | Número de días  No comió  No sé / No recuerdo | Miembros del hogar mayores de 15 años |
| ¿Cuántas porciones de ensalada de vegetales comiste por día?  Tenga en cuenta que una porción es equivalente a 4 cucharas | Número de porciones  Número de cucharas  No sé / No recuerdo_ | Miembros del hogar mayores de 15 años |
| **PREVENCIÓN DE CANCER** |  | Miembros del hogar mayores de 15 años |
| ¿Sabe qué es el cáncer? | Si  No  No sabe/No recuerda |  |
| ¿Considera que se puede prevenir el cáncer? | Si  No  No sabe/No recuerda | Miembro del hogar del sexo femenino mayor de 15 años y menor de 60 años |
| ¿Alguna vez en su vida ha oído hablar del cáncer de cuello uterino, también llamado cáncer cervical? | Si  No  No sabe/No recuerda | Miembro del hogar del sexo femenino mayor de 15 años y menor de 60 años |
| ¿Alguna vez en su vida ha oído hablar del virus del papiloma humano? | Si  No  No sabe/No recuerda | Miembro del hogar del sexo femenino mayor de 15 años y menor de 60 años |
| ¿Cree que el virus del papiloma humano puede causar cáncer de cuello uterino, también llamado cáncer cervical? | Si  No  No sabe/No recuerda | Miembro del hogar del sexo femenino mayor de 15 años y menor de 60 años |
| En los últimos 2 años, es decir, ¿se ha realizado algún examen o chequeo para descartar cualquier tipo de cáncer? | Si  No  No sabe/No recuerda | Miembro del hogar del sexo femenino entre 40 y 59 años |
| ¿Dónde le realizaron dicho examen o chequeo | Hospital publico  Centro/puesto de salud publico  Hospital de Essalud  Policlínico/posta de Essalud  Fuerzas armadas / Policía  Clínica/consultorio privado  Clínica/posta de ONG  Hospital de iglesia  Campaña del ministerio  Campaña de Essalud  Otras campañas  Otros  No sé / No recuerdo | Miembro del hogar del sexo femenino entre 40 y 59 años |
| ¿Alguna vez en su vida un médico u otro profesional de salud le ha realizado un examen físico de mama? | Si  No  No sabe/No recuerda | Miembro del hogar del sexo femenino entre 30 y 59 años |
| ¿Hace cuánto tiempo le realizaron la última vez el examen físico de mama? | En "meses", si <2 años __________________  En "años", si 2 o más años ______________________  No sé / no recuerdo ____________________ | Miembro del hogar del sexo femenino entre 30 y 59 años |
| ¿Alguna vez en su vida un médico u otro profesional de salud le ha realizado la prueba de papanicolaou? | Si  No  No sabe/No recuerda | Miembro del hogar del sexo femenino entre 30 y 59 años |
| ¿Hace cuánto tiempo le realizaron la última vez la prueba de papanicolao? | En "meses", si <2 años __________________  En "años", si 2 o más años ______________________  No sé / no recuerdo ____________________ | Miembro del hogar del sexo femenino entre 30 y 59 años |
| ¿Recogió o conoció los resultados de la prueba de Papanicolau? | Si  No  No sabe/No recuerda | Miembro del hogar del sexo femenino entre 30 y 59 años |
| **TUBERCULOSIS** |  |  |
| ¿Tiene usted actualmente una tos con flema? | Si  No | Miembro del hogar mayor de 15 años |
| ¿Hace cuánto tiempo tiene usted tos con flema? | Tiempo | Miembro del hogar mayor de 15 años |
| ¿Conoce o ha oído hablar de una enfermedad llamada tuberculosis o TBC? | Si  No  No sabe/No recuerda | Miembro del hogar mayor de 15 años |
| Usted cree que la tuberculosis se contagia?... :   1. ¿Cuándo una persona enferma tose o estornuda cerca de otra persona? 2. ¿Cuándo una persona enferma habla de cerca con otra persona? 3. ¿Cuándo una persona enferma toca a otra persona? 4. ¿Cuándo una persona enferma comparte comida o utensilios con otra persona? | Si  No  No sabe/No recuerda | Miembro del hogar mayor de 15 años |
| ¿Puede curarse la tuberculosis? | Si  No  No sabe/No recuerda | Miembro del hogar mayor de 15 años |
| **VIH/SIDA** |  |  |
| 1. Usted ha oído hablar de:   a. La Infección por VIH?  B. ¿Una enfermedad llamada SIDA? | Si  No  No sabe/No recuerda | Miembro del hogar mayor de 15 años |
| En este último año, ¿le han hecho la prueba para saber si tiene el virus que causa el SIDA (VIH)? | Si  No  No sabe/No recuerda | Miembro del hogar mayor de 15 años |
| Usted conoció los resultados del examen de laboratorio? | Si  No  No sabe/No recuerda | Miembro del hogar mayor de 15 años |
| ¿Cree usted que las personas tienen menos riesgo de adquirir el virus que causa el SIDA (VIH), si tienen una sola pareja sexual que no esté infectada y que no tenga otras parejas? | Si  No  No sabe/No recuerda | Miembro del hogar mayor de 15 años |
| ¿Es posible que una persona de aspecto saludable esté infectada con el virus que causa el SIDA? | Si  No  No sabe/No recuerda | Miembro del hogar mayor de 15 años |
| ¿Cree usted que las personas pueden adquirir el virus que causa el SIDA (VIH) por dar un abrazo, beso o caricias a una persona infectada? | Si  No  No sabe/No recuerda | Miembro del hogar mayor de 15 años |
| ¿Cree usted que las personas pueden adquirir el virus que causa el SIDA (VIH) por la picadura de un mosquito? | Si  No  No sabe/No recuerda | Miembro del hogar mayor de 15 años |
| ¿Cree usted que las personas pueden adquirir el virus que causa el SIDA (VIH) compartiendo alimentos o utensilios como: cubiertos, vasos, etc. con una persona infectada? | Si  No  No sabe/No recuerda | Miembro del hogar mayor de 15 años |
| ¿Cree usted que las personas que usan condón cada vez que tienen relaciones sexuales tienen menos riesgo de adquirir el virus que causa el SIDA (VIH)? | Si  No  No sabe/No recuerda | Miembro del hogar mayor de 15 años |
| **CONTRACEPCIÓ**N |  |  |
| Ahora me gustaría hablar con usted acerca de la planificación familiar, es decir, sobre las diferentes formas o métodos que una pareja puede usar para retrasar (detener más adelante) o evitar el embarazo. Entonces les diré una serie de métodos anticonceptivos y me dirán si los conocen o si los han usado alguna vez | Esterilización femenina (ligadura de trompas) (Conocimiento / Uso)  Esterilización masculina (vasectomía) (Conocimiento / Uso)  Píldora (Conocimiento / Uso)  "T de Cobre" (Conocimiento / Uso)  Inyección de anticonceptivos (Conocimiento / Uso)  Implantes, norplant (Conocimiento / Uso)  Condón (Conocimiento / Uso)  Preservativo o condón femenino (Conocimiento / Uso)  Espuma, jalea, óvulos (métodos vaginales) (Conocimiento / Uso)  Métodos de lactancia materna exclusiva (Conocimiento / Uso)  Abstinencia periódica (Conocimiento / Uso)  Retiro (conocimiento / uso)  Píldora del día siguiente (Conocimiento / Uso)  Otros Métodos _____________________ | Miembro del hogar de sexo femenino mayor de 15 años |
| ¿Alguna vez usted o su pareja han usado algo para posponer o evitar el embarazo? | Si  No | Miembro del hogar de sexo femenino mayor de 15 años |
| ¿Está usted o su pareja haciendo algo o usando algún método para retrasar o evitar quedar embarazada? | Si  No | Miembro del hogar de sexo femenino mayor de 15 años |
| ¿Qué método está usando? | Pregunta abierta | Miembro del hogar de sexo femenino mayor de 15 años |
| **EMBARAZO, PARTO, PUERPERIO Y LACTANCIA** |  |  |
| Cuando quedó embarazada de ______, ¿quería quedar embarazada entonces, quería esperar más tiempo o no quería tener más hijos? | Queria quedar embarazada  Esperar mas tiempo  No queria tener más hijos | Miembro del hogar femenino con por lo menos 1 hijo |
| ¿Cuánto tiempo más te hubiera gustado esperar? | Número de tiempo | Miembro del hogar femenino con por lo menos 1 hijo |
| Cuando estuvo embarazada de ____, ¿tuvo cuidado prenatal o controles prenatales? | Si  No  No recuerda | Miembro del hogar femenino con por lo menos 1 hijo |
| ¿Dónde se controló? | Hospital publico  Centro/puesto de salud publico  Hospital de Essalud  Policlínico/posta de Essalud  Fuerzas armadas / Policía  Clínica/consultorio privado  Clínica/posta de ONG  Hospital de iglesia  Campaña del ministerio  Campaña de Essalud  Otras campañas  Otros  No sé / No recuerdo | Miembro del hogar femenino con por lo menos 1 hijo |
| ¿Cuántos meses de embarazo tuvo cuando realizó su primer examen prenatal? | Número de meses | Miembro del hogar femenino con por lo menos 1 hijo |
| Durante los exámenes prenatales, ¿hizo alguna de las siguientes pruebas? | Peso  Medir el vientre  Tomar la presión arterial  Examen de orina  Prueba de sangre  Escucharon el latido del corazón del bebé  Estudios para descartar la sífilis  Estudios para descartar el VIH / SIDA | Miembro del hogar femenino con por lo menos 1 hijo |
| ¿Te explicaron las complicaciones que pueden ocurrir durante el embarazo? | Si  No  No recuerdo | Miembro del hogar femenino con por lo menos 1 hijo |
| ¿Le dijeron dónde ir si tuviera alguna de estas complicaciones? | Si  No  No recuerdo | Miembro del hogar femenino con por lo menos 1 hijo |
| Durante el ___________ embarazo, ¿estuvo afiliado al "Seguro Integral de Salud o Materno Infantil"? | Si  No  No recuerdo | Miembro del hogar femenino con por lo menos 1 hijo |
| Cuando estuvo embarazada, ¿tuvo alguna vacuna en su brazo o nalga para prevenir al bebé del tétanos, es decir, las convulsiones después del nacimiento? | Si  No  No recuerdo | Miembro del hogar femenino con por lo menos 1 hijo |
| ¿Recibió alguna vacuna contra el tétanos antes del embarazo? | Si  No  No recuerdo | Miembro del hogar femenino con por lo menos 1 hijo |
| ¿Dónde dio a luz? | En su propia casa  Hospital público  Posta de salud  Policlinico  Clinica privada  ONG  Hospital de la iglesia | Miembro del hogar femenino con por lo menos 1 hijo |
| ¿Por qué no fue a un hospital, un centro de salud o un puesto de salud en el Ministerio de Salud para dar a luz? | No existe en la localidad  Estaba muy lejos  No había personal  Estaba afiliada a otro servicio de salud  Personal daba malos tratos  No había privacidad en la atención  Mi esposo / familiar se opuso  No era higiénico  No confío en la atención  El personal es descuidado  Congestión del servicio  No tenía dinero  No querían atenderme  yo estaba en el camino hacia el centro, pero el parto se adelantó  Otro | Miembro del hogar femenino con por lo menos 1 hijo |
| _____________ nació por cesárea? | Si  No | Miembro del hogar femenino con por lo menos 1 hijo |
| ¿Se programó la cesárea? | Si  No | Miembro del hogar femenino con por lo menos 1 hijo |
| Cuando nació tuvo algunas de estas complicaciones | Trabajo de parto prolongado, es decir, las contracciones fuertes y regulares duraron más de 12 horas?  Sangrado excesivo después del parto  Fiebre alta con sangrado vaginal que olía mal?  Convulsiones no causadas por fiebre?  Alguna otra complicación? | Miembro del hogar femenino con por lo menos 1 hijo |
| Después del nacimiento de _____________ ¿tuvo algún control o chequeo médico? | Si  No | Miembro del hogar femenino con por lo menos 1 hijo |
| Durante los 40 días posteriores al parto? Si alguna de estas complicaciones | sangrado abundante de la vagina  Desmayo o pérdida del conocimiento  Fiebre alta o escalofríos  Infección de mama/senos  Dolor o ardor al orinar?  Flujos o líquidos vaginales?  Pérdida involuntaria de orina | Miembro del hogar femenino con por lo menos 1 hijo |
| ¿Cuánto peso tiene el niño / niña? ___________________ | Peso | Miembro del hogar femenino con por lo menos 1 hijo |
| Durante el primer mes después de que dio a luz __________, ¿tuvo algún chequeo médico o chequeo médico en el bebé? | Si  No | Miembro del hogar femenino con por lo menos 1 hijo |
| ¿Dónde recibiste este chequeo? | Hospital publico  Centro/puesto de salud publico  Hospital de Essalud  Policlínico/posta de Essalud  Fuerzas armadas / Policía  Clínica/consultorio privado  Clínica/posta de ONG  Hospital de iglesia  Campaña del ministerio  Campaña de Essalud  Otras campañas  Otros  No sé / No recuerdo | Miembro del hogar femenino con por lo menos 1 hijo |
| ¿Le dio leche materna a ______________? | Si  No | Miembro del hogar femenino con por lo menos 1 hijo |
| ¿Por qué no le dio pecho a _________? | Madre enferma / débil  Hijo enfermo / débil  El niño murió  Problema de succión del niño  Problema de succión del pezón  No tenía leche  Madre trabajando  El niño se negó  Otro_________ | Miembro del hogar femenino con por lo menos 1 hijo |
| ¿Cuánto tiempo después de su nacimiento comenzó a amamantarlo? | Tiempo | Miembro del hogar femenino con por lo menos 1 hijo |
| Durante los tres primeros días de parto, ¿le dio a _______________ algo más que tomar leche materna? | SI  No | Miembro del hogar femenino con por lo menos 1 hijo |
| ¿Qué le dio? | -Cualquier leche que no sea leche materna  Agua sola  glucosa/dextrosa  Agua azucarada  Jugo de fruta  Fórmula para bebés  Té / Infusiones  Miel  Otro | Miembro del hogar femenino con por lo menos 1 hijo |
| ¿Se puso en contacto piel con piel con el bebé cuando nació? | Si  No | Miembro del hogar femenino con por lo menos 1 hijo |
| ¿Cuántos meses le diste pecho (leche materna)? _________ | Meses | Miembro del hogar femenino con por lo menos 1 hijo |
| Por qué dejó de darle de pecho | Madre enferma / débil  Hijo enfermo / débil  El niño murió  Problema de succión del niño  Problema de succión del pezón  No tenía leche  Madre trabajando  El niño se negó  Edad de destete  Quedó embarazada  Comenzó a usar anticonceptivos  Niño sólo quería pecho  Otro_________ | Miembro del hogar femenino con por lo menos 1 hijo |
| **SALUD OCULAR** |  |  |
| ¿A (NOMBRE) alguna vez en su vida, un médico u otro personal de salud le ha evaluado o medido la vista con esta cartilla? (presentar la cartilla de Snell) | Si  No  No se / No recuerdo | Cada miembro del hogar niñas o niños de 3 a 11 años |
| Hace cuánto tiempo fue la última vez que le evaluaron o midieron la vista? | En "meses", si <2 años  En "años", si 2 o más años  No sé / no recuerdo | Cada miembro del hogar niñas o niños de 3 a 11 años |
| ¿Dónde le evaluaron o midieron la vista la última vez? | Hospital publico  Centro/puesto de salud publico  Hospital de Essalud  Policlínico/posta de Essalud  Fuerzas armadas / Policía  Clínica/consultorio privado  Clínica/posta de ONG  Hospital de iglesia  Campaña del ministerio  Campaña de Essalud  Otras campañas  Otros  No sé / No recuerdo | Cada miembro del hogar niñas o niños de 3 a 11 años |
| ¿A (NOMBRE) le han diagnosticado un problema de visión y además le han indicado el uso de lentes o anteojos? | Si  No | Cada miembro del hogar niñas o niños de 3 a 11 años |
| Dónde le diagnosticaron el problema de visión? | Hospital publico  Centro/puesto de salud publico  Hospital de Essalud  Policlínico/posta de Essalud  Fuerzas armadas / Policía  Clínica/consultorio privado  Clínica/posta de ONG  Hospital de iglesia  Campaña del ministerio  Campaña de Essalud  Otras campañas  Otros  No sé / No recuerdo | Cada miembro del hogar niñas o niños de 3 a 11 años |
| ¿Hace cuánto tiempo le indicaron el uso de lentes o anteojos a (NOMBRE)? | En "meses", si <2 años  En "años", si 2 o más años  No sé / no recuerdo | Cada miembro del hogar niñas o niños de 3 a 11 años |
| ¿(NOMBRE) está usando lentes o anteojos para mejorar su problema de visión? | Si  No | Cada miembro del hogar niñas o niños de 3 a 11 años |
| ¿Por qué motivo (NOMBRE) no está usando lentes o anteojos para corregir su problema de visión? | No compró por el precio  Se rompieron  No le gustan  Lo molestan en el colegio  Otro___  No sabe/ no recuerda | Cada miembro del hogar niñas o niños de 3 a 11 años |
| ¿Por qué motivo (NOMBRE) no usa lentes o anteojos siempre? | Descuido  Le incomodan  No le gustan  Le molestan en el colegio  Otro  No sabe/no recuerda | Cada miembro del hogar niñas o niños de 3 a 11 años |
| ¿(NOMBRE) ve televisión muy de cerca, es decir, a menos de 30 centímetros? | Si  No  No ve televisión  No sabe/ No recuerda | Cada miembro del hogar niñas o niños de 3 a 11 años |
| Cuando (NOMBRE) está en un lugar sin techo, haga o no haga calor, ¿Con qué frecuencia usa gorro con visera, sombrero o lentes de sol, nunca, a veces o siempre? | Nunca  A veces  Siemore | Cada miembro del hogar niñas o niños de 3 a 11 años |
| ¿(NOMBRE) está usando lentes o anteojos, a veces, casi siempre o siempre? | A veces  Casi siemore  Siempre | Cada miembro del hogar niñas o niños de 3 a 11 años |
| ¿Considera usted que (NOMBRE) tiene buena iluminación en el ambiente donde lee? | Si  No  No sabe leer | Cada miembro del hogar niñas o niños de 6 a 11 años |
| ¿(NOMBRE) acostumbra leer un texto mientras está caminando, en la balsa o bote? | Si  No  No sabe leer | Cada miembro del hogar niñas o niños de 6 a 11 años |
| **SALUD MENTAL** |  |  |
| En las dos últimas semanas, usted ha experimentado molestias o problemas como:   1. ¿Pocas ganas o interés de hacer las cosas? (DE SER NECESARIO LEA: Es decir, no disfruta sus actividades cotidianas) 2. ¿Se siente desanimado, deprimido, triste o sin esperanzas? 3. ¿Problemas para dormir o mantenerse dormido, o en dormir demasiado? 4. ¿Sentirse cansado o tener poca energía sin motivo que lo justifique? 5. ¿Poco apetito o comer en exceso? 6. ¿Dificultad para poner atención o concentrarse en las cosas que hace? (DE SER NECESARIO LEA: Como leer el periódico, ver televisión, escuchar atentamente la radio o conversar con otras personas) | Para nada  Varios días (1 a 6 días)  La mayoría de días (7 a 11 días)  Casi todos los días (12 a + días) | Cada miembro del hogar mayor de 15 años |
| ¿Estas molestias o problema cuánto le han dificultado para realizar su trabajo, sus actividades en casa o llevarse bien con otras personas, mucho, poco o nada? | Mucho  Poco  Nada | Cada miembro del hogar mayor de 15 años |
| En este último año, usted ha tenido molestias o problemas mencionados anteriormente tales como: sentirse con poco interés, triste, con problemas para dormir, desanimado, cansado sin motivo, ¿desconcentrado o con poco apetito? | Si  No | Cada miembro del hogar mayor de 15 años |
| En el último año, ¿ha recibido tratamiento de algún profesional de la salud por depresión, tristeza, desánimo, falta de interés o irritabilidad? | Si  No  No sabe/no recuerda | Cada miembro del hogar mayor de 15 años |
| ¿Dónde recibió tratamiento? ¿En algún otro lugar? | Hospital publico  Centro/puesto de salud publico  Hospital de Essalud  Policlínico/posta de Essalud  Fuerzas armadas / Policía  Clínica/consultorio privado  Clínica/posta de ONG  Hospital de iglesia  Campaña del ministerio  Campaña de Essalud  Otras campañas  Otros  No sé / No recuerdo | Cada miembro del hogar mayor de 15 años |
| En el último mes, ¿(NOMBRE) asistió a la escuela o colegio? | Si  No  No se / No recuerdo | Cada miembro del hogar niñas o niños de 6 a 11 años |
| En el último mes, ¿Cuántas veces (NOMBRE) fue golpeada(o) por un profesor o por una profesora? | Nunca  Rara vez (1 a 2 veces)  A menudo (6 veces a más)  No sabe/No recuerda | Cada miembro del hogar niñas o niños de 6 a 11 años |
| En el último mes, ¿Cuántas veces (NOMBRE) fue golpeada(o) por otro estudiante? | Nunca  Rara vez (1 a 2 veces)  A menudo (6 veces a más)  No sabe/No recuerda | Cada miembro del hogar niñas o niños de 6 a 11 años |
| ¿(NOMBRE) tiene confianza para contarle sus cosas a algún profesor o profesora de su escuela o colegio? | Si  No  No se / No recuerdo | Cada miembro del hogar niñas o niños de 6 a 11 años |
| ¿Durante este año, alguna vez (NOMBRE) no quiso ir a su escuela o colegio por miedo a que alguien le pegue ? | Si  No  No se / No recuerdo | Cada miembro del hogar niñas o niños de 6 a 11 años |
| **SALUD BUCAL** |  |  |
| ¿Alguna vez en su vida usted, ha sido atendida/o en un servicio dental o por un odontólogo? | Si  No  No se / No recuerdo | Cada miembro del hogar mayor de 15 años |
| ¿Hace cuánto tiempo fue la última atención? | Número en meses o años  No recuerdo | Cada miembro del hogar mayor de 15 años |
| ¿Dónde le atendieron por última vez? | Hospital publico  Centro/puesto de salud publico  Hospital de Essalud  Policlínico/posta de Essalud  Fuerzas armadas / Policía  Clínica/consultorio privado  Clínica/posta de ONG  Hospital de iglesia  Campaña del ministerio  Campaña de Essalud  Otras campañas  Otros  No sé / No recuerdo | Cada miembro del hogar mayor de 15 años |
| ¿Alguna vez ha experimentado dolor de muelas o de dientes que ha persistido durante más de una semana? | Si  No  No se / No recuerdo | Cada miembro del hogar mayor de 15 años |
| ¿Alguna vez en su vida, (NOMBRE) ha sido atendida/o en un servicio dental o por un odontólogo? | Si  No  No se / No recuerdo | Cada miembro del hogar menor a 15 años y/o su cuidador/persona responsable |
| ¿Hace cuánto tiempo fue la última atención? | Número en meses o años  No recuerdo | Cada miembro del hogar menor a 15 años y/o su cuidador/persona responsable |
| ¿Dónde le atendieron por última vez? | Hospital publico  Centro/puesto de salud publico  Hospital de Essalud  Policlínico/posta de Essalud  Fuerzas armadas / Policía  Clínica/consultorio privado  Clínica/posta de ONG  Hospital de iglesia  Campaña del ministerio  Campaña de Essalud  Otras campañas  Otros  No sé / No recuerdo | Cada miembro del hogar menor a 15 años y/o su cuidador/persona responsable |
| En el último año, ¿usted ha recibido información sobre el cuidado e higiene de los dientes, lengua y boca de las niñas y niños de una persona o medio de comunicación? | Si  No  No se / No recuerdo | Cada miembro del hogar menor a 15 años y/o su cuidador/persona responsable |
| ¿Dónde recibió́ la información? | Ministerio de Salud  ESSALUD  FFAA o FFPP  Municipalidad  Sector Privado  ONG/Iglesia  Organizaciones de base  Medios de comunicación  Instituciones educativas  Familiares, vecinos, amigos  Otros | Cada miembro del hogar menor a 15 años y/o su cuidador/persona responsable |
| ¿(NOMBRE) se lava los dientes con cepillo dental? | Si  No | Cada miembro del hogar menor a 15 años y/o su cuidador/persona responsable |
| ¿(NOMBRE) se cepilla los dientes todos los días? | Si  No  No se / No recuerdo | Cada miembro del hogar menor a 15 años y/o su cuidador/persona responsable |
| ¿(NOMBRE) cuántas veces al día se cepilla los dientes? | Una vez al día  Dos veces al día  Tres veces al día  Cuatro o más veces al día | Cada miembro del hogar menor a 15 años y/o su cuidador/persona responsable |
| ¿Cuánto tiempo tiene el cepillo que (NOMBRE) usa para lavarse los dientes? | Meses  Uno o más años  No sabe / No recuerda | Cada miembro del hogar menor a 15 años y/o su cuidador/persona responsable |
| ¿Cuántas personas usan el cepillo dental de (NOMBRE)? | Sólo el niño o la niña  Más de una persona | Cada miembro del hogar menor a 15 años y/o su cuidador/persona responsable |
| ¿(NOMBRE) usa crema dental cuando se cepilla los dientes? | Si  No | Cada miembro del hogar menor a 15 años y/o su cuidador/persona responsable |
