# Supplementary material for: Health need assessment in an indigenous high-altitude population living on an island in Lake Titicaca, Perú
Source: Int J Equity Health. 2019 Jun 18;18:94. doi: 10.1186/s12939-019-0993-3 (PMC6582488; doi:10.1186/s12939-019-0993-3)
Supplement: Supplementary file 2 — Complementary bivariate analysis of health needs. (DOCX 24 kb) [file 12939_2019_993_MOESM2_ESM.docx]

**APPENDIX 2: BIVARIATE ANALYSIS**

**Table 14:** Association between health needs and gender

| Characteristic | Female % (95% CI) | Male % (95% CI) | P-value | |
| --- | --- | --- | --- | --- |
| **Adults** |  |  |  |  |
| *Non-communicable diseases and risk factors* |  |  |  |  |
| Blood pressure measurement in the last year | 39.4 (31.2-48.1) | 33.7 (23.9-45.1) | 0.397 |  |
| Self-report of a diagnosis of high blood pressure | 6.6 (3.3-12.7) | 4.9 (1.9-12.5) | 0.634 |  |
| Blood glucose measurement in the last year | 16.3 (10.5-24.4) | 17.4 (10.9-26.8) | 0.797 |  |
| Self-report of a diagnosis of diabetes mellitus | 2.5 (0.1-7.6) | 0.0 (N/A) | 0.163 |  |
| Smoking in the last year | 3.8 (1.6-8.8) | 26.7 (18.4-37.0) | <0.001 |  |
| Smoking in the last month | 2.3 (0.7-6.9) | 11.1 (6.1-19.3) | 0.003 |  |
| Lifetime prevalence of alcohol use | 31.3 (24.1-39.5) | 52.2 (41.6-62.6) | <0.001 |  |
| Alcohol use in the last month | 7.8 (4.2-14.2) | 12.5 (6.8-21.8) | 0.160 |  |
| Fruit consumption in the last week | 87.0 (79.2-92.2) | 93.5 (74.3-89.9) | 0.366 |  |
| Vegetable consumption in the last week | 93.9 (87.3-97.2) | 96.7 (90.2-98.9) | 0.317 |  |
| *Eye health* |  |  |  |  |
| Lifetime prevalence of visual acuity testing | 18.4 (12.6-26.1) | 30.6 (21.6-41.4) | 0.028 |  |
| Difficulty seeing/recognising a face within 6 metres in the last year | 44.1 (35.8-52.8) | 44.9 (35.6-54.6) | 0.895 |  |
| *Oral health* |  |  |  |  |
| Lifetime prevalence of dental exam | 63.0 (54.3-70.8) | 65.0 (54.4-74.2) | 0.741 |  |
| Tooth pain persisting more than one week | 53.3 (44.6-61.9) | 57.7 (47.2-67.6) | 0.466 |  |
| *Communicable diseases* |  |  |  |  |
| Productive cough in the last 2 weeks | 14.4 (9.4-21.4) | 9.9 (5.1-18.2) | 0.313 |  |
| Heard about tuberculosis | 52.9 (43.4-62.2) | 61.8 (50.8-71.8) | 0.161 |  |
| Heard about HIV/AIDS | 20.2 (15.6-25.8) | 20.1 (14.7-26.9) | 0.984 |  |
| *Depression* |  |  |  |  |
| Self-report of anhedonia or sadness | 28.6 (22.9-35.0) | 21.3 (15.4-28.7) | 0.084 |  |
| **Children** |  |  |  |  |
| *Eye health* |  |  |  |  |
| Lifetime prevalence of visual acuity testing | 63.3 (45.6-78.1) | 57.7 (37.5-75.6) | 0.654 |  |
| Self-report of a diagnosis of visual impairment or prescription of glasses | 10.5 (2.3-36.6) | 30.8 (11.7-59.8) | 0.160 |  |
| *Oral health* |  |  |  |  |
| Lifetime prevalence of dental exam | 61.9 (47.2-74.7) | 61.0 (47.3-73.2) | 0.924 |  |
| Daily brushing of teeth | 67.9 (49.8-81.8) | 72.7 (57.8-83.9) | 0.607 |  |
| *Physical violence* |  |  |  |  |
| Corporal punishment by a teacher in the last month | 20.8 (8.7-42.0) | 15.8 (4.8-40.9) | 0.677 |  |
| Physical violence by peer in school | 17.4 (6.5-38.8) | 36.8 (18.8-59.5) | 0.149 |  |

**Table 15:** Association between health needs and age

| Characteristic (among adults) | Young adults (18-34 yrs) % (95% CI) | Middle-aged adults (35-59 yrs) % (95% CI) | Older adults  (≥60 yrs) % (95% CI) | P-value |
| --- | --- | --- | --- | --- |
| *Non-communicable diseases and risk factors* |  |  |  |  |
| Blood pressure measurement in the last year | 34.5 (22.5-48.8) | 30.2 (21.3-40.9) | 48.2 (34.8-61.9) | 0.109 |
| Self-report of a diagnosis of high blood pressure | 3.7 (0.9-14.0) | 5.6 (2.3-12.7) | 7.6 (2.8-18.9) | 0.693 |
| Blood glucose measurement in the last year | 22.0 (12.6-35.6) | 13.8 (7.7-23.5) | 14.0 (6.7-26.9) | 0.387 |
| Self-report of a diagnosis of diabetes mellitus | 3.8 (0.9-14.2) | 1.1 (0.2-7.4) | 0 (N/A) | 0.280 |
| Smoking in the last year | 8.5 (3.6-18.9) | 12.0 (7.0-19.7) | 19.3 (10.8-32.2) | 0.203 |
| Smoking in the last month | 3.4 (0.8-12.8) | 5.0 (2.1-11.5) | 8.8 (3.2-22.1) | 0.466 |
| Lifetime prevalence of alcohol use | 35.6 (24.5-48.4) | 46.0 (35.0-57.4) | 33.9 (23.3-46.6) | 0.252 |
| Alcohol use in the last month | 7.6 (2.9-18.5) | 14.3 (7.5-25.5) | 4.4 (6.0-16.3) | 0.164 |
| Fruit consumption in the last week | 93.2 (83.2-97.5) | 84.0 (73.8-90.7) | 80.7 (65.7-90.1) | 0.147 |
| Vegetable consumption in the last week | 96.6 (87.2-99.2) | 95.0 (86.9-98.2) | 93.0 (82.4-97.4) | 0.662 |
| *Eye health* |  |  |  |  |
| Lifetime prevalence of visual acuity testing | 40.0 (27.8-53.6) | 13.0 (7.4-21.8) | 21.1 (11.7-35.0) | 0.001 |
| Difficulty seeing/recognising a face within 6 metres in the last year | 23.3 (13.7-36.9) | 41.0 (31.2-51.6) | 84.2 (72.3-91.6) | <0.001 |
| *Oral health* |  |  |  |  |
| Lifetime prevalence of dental exam | 64.4 (50.3-76.4) | 68.0 (57.7-76.8) | 56.1 (41.6-69.7) | 0.361 |
| Tooth pain persising more than one week | 52.5 (39.3-65.4) | 53.0 (41.8-63.9) | 64.9 (50.7-76.9) | 0.319 |
| *Communicable diseases* |  |  |  |  |
| Productive cough in the last 2 weeks | 13.3 (6.6-25.0) | 11.0 (6.3-18.6) | 8.8 (3.6-19.9) | 0.744 |
| Heard about tuberculosis | 67.9 (52.8-80.0) | 60.2 (48.0-71.3) | 34.2 (20.5-51.3) | 0.010 |
| Heard about HIV/AIDS | 58.3 (44.0-71.4) | 28.0 (19.9-37.9) | 8.8 (3.7-19.6) | <0.001 |
| *Cancer* |  |  |  |  |
| Knowledge of cancer prevention (among women) | 23.3 (14.5-35.3) | 15.0 (9.3-23.4) | 0 (N/A) | <0.001 |
| *Depression* |  |  |  |  |
| Self-report of anhedonia or sadness | 41.7 (29.1-55.5) | 39.0 (28.9-50.1) | 40.4 (32.9-47.8) | 0.953 |

**Table 16:** Association between health needs and level of education

| Characteristic (among adults) | Primary school % (95% CI) | Secondary school % (95% CI) | Superior education % (95% CI) | P-value |
| --- | --- | --- | --- | --- |
| *Non-communicable diseases and risk factors* |  |  |  |  |
| Blood pressure measurement in the last year | 31.6 (22.1-42.9) | 29.5 (19.6-41.7) | 57.1 (22.6-85.9) | 0.341 |
| Self-report of a diagnosis of high blood pressure | 2.9 (70.7-11.1) | 2.7 (0.7-10.4) | 28.6 (7.0-67.9) | 0.004 |
| Blood glucose measurement in the last year | 16.9 (9.7-27.7) | 17.7 (10.1-29.4) | 42.9 (14.1-77.4) | 0.260 |
| Self-report of a diagnosis of diabetes mellitus | 0 (N/A) | 2.7 (0.7-10.4) | 16.7 (2.2-63.9) | 0.018 |
| Smoking in the last year | 14.1 (8.2-23.3) | 8.8 (3.8-18.9) | 57.1 (22.6-85.9) | 0.003 |
| Smoking in the last month | 6.4 (2.7-14.5) | 3.8 (1.2-11.1) | 28.6 (7.1-67.9) | 0.031 |
| Lifetime prevalence of alcohol use | 44.9 (34.4-55.8) | 47.5 (35.8-59.5) | 71.4 (32.1-93.0) | 0.411 |
| Alcohol use in the last month | 12.5 (5.9-24.7) | 12.3 (6.1-23.3) | 14.3 (1.9-58.9) | 0.990 |
| Fruit consumption in the last week | 85.9 (74.2-92.8) | 90.1 (81.6-94.9) | 83.3 (36.2-97.8) | 0.677 |
| Vegetable consumption in the last week | 98.7 (91.2-99.8) | 95.1 (87.4-98.2) | 1.00 (N/A) | 0.367 |
| *Eye health* |  |  |  |  |
| Lifetime prevalence of visual acuity testing | 16.5 (9.3-27.4) | 33.0 (23.3-44.3) | 28.6 (7.1-67.9) | 0.052 |
| Difficulty seeing/recognising a face within 6 metres in the last year | 41.8 (30.5-54.0) | 30.8 (21.8-41.5) | 42.9 (14.1-77.4) | 0.332 |
| *Oral health* |  |  |  |  |
| Lifetime prevalence of dental exam | 60.8 (49.4-71.1) | 66.7 (55.3-76.4) | 83.3 (36.2-97.8) | 0.460 |
| Tooth pain perstising more than one week | 50.6 (38.6-62.6) | 56.7 (45.4-67.3)) | 0.50 (16.5-83.5) | 0.722 |
| *Communicable diseases* |  |  |  |  |
| Productive cough in the last 2 weeks | 11.5 (6.0-20.9) | 11.1 (5.8-20.2) | 14.3 (1.9-58.8) | 0.968 |
| Heard about tuberculosis | 53.3 (39.5-66.7) | 77.5 (64.7-86.6) | 83.3 (36.1-97.8) | 0.016 |
| Heard about HIV/AIDS | 17.3 (11.8-24.7) | 35.8 (27.0-45.7) | 71.4 (32.2-92.9) | <0.001 |
| *Cancer* |  |  |  |  |
| Knowledge of cancer prevention (among women) | 6.8 (3.6-12.5) | 12.5 (7.6-20.0) | 42.9 (14.1-77.4) | 0.007 |
| *Depression* |  |  |  |  |
| Self-report of anhedonia or sadness | 19.6 (13.7-27.1) | 28.3 (20.3-38.0) | 28.6 (7.1-67.8) | 0.251 |
